# Increased bone mineral density for 1 year of romosozumab, vs placebo, followed by 2 years of denosumab in the Japanese subgroup of the pivotal FRAME trial and extension

**DOI:** 10.1007/s11657-019-0608-z

**Published:** 2019-06-05

**Authors:** Akimitsu Miyauchi, Rajani V. Dinavahi, Daria B. Crittenden, Wenjing Yang, Judy C. Maddox, Etsuro Hamaya, Yoichi Nakamura, Cesar Libanati, Andreas Grauer, Junichiro Shimauchi

**Affiliations:** 1 Miyauchi Medical Center, Osaka, Japan; 2 Amgen Inc., Thousand Oaks, CA USA; 3 Amgen Astellas BioPharma K.K., Tokyo, Japan; 4 UCB Pharma, Brussels, Belgium

**Keywords:** Romosozumab, Denosumab, Japanese, Bone mineral density, Fracture

## Abstract

***Summary*:**

Romosozumab, which binds sclerostin, rebuilds the skeletal foundation before transitioning to antiresorptive treatment. This subgroup analysis of an international, randomized, double-blind study in postmenopausal women with osteoporosis showed efficacy and safety outcomes for romosozumab followed by denosumab in Japanese women were generally consistent with those for the overall population.

**Purpose:**

In the international, randomized, double-blind, phase 3 FRActure study, in postmenopausal woMen with ostEoporosis (FRAME; NCT01575834), romosozumab followed by denosumab significantly improved bone mineral density (BMD) and reduced fracture risk. This report evaluates Japanese women in FRAME.

**Methods:**

Postmenopausal women with osteoporosis (T-score − 3.5 to − 2.5 at total hip or femoral neck) received romosozumab 210 mg or placebo subcutaneously monthly for 12 months, then each group received denosumab 60 mg subcutaneously every 6 months for 24 months. The key endpoint for Japanese women was BMD change. Other endpoints included new vertebral, clinical, and nonvertebral fracture; the subgroup analysis did not have adequate power to demonstrate statistically significant reductions.

**Results:**

Of 7180 enrolled subjects, 492 (6.9%) were Japanese (247 romosozumab, 245 placebo). BMD increases from baseline were greater (*P* < 0.001) for romosozumab-to-denosumab than placebo-to-denosumab at the lumbar spine (36 months, 12.7%), total hip (4.2%), and femoral neck (4.1%). Fracture risk was lower through 36 months for romosozumab-to-denosumab vs placebo-to-denosumab for new vertebral (1.7% vs 4.5%; relative risk reduction (RRR) 63%, *P* = 0.070), clinical (3.2% vs 7.3%; RRR 53%, *P* = 0.072), nonvertebral (2.8% vs 6.1%; RRR 50%, *P* = 0.12), and all other fracture types evaluated. Rates of adverse events and positively adjudicated serious cardiovascular events were generally balanced between groups.

**Conclusions:**

Efficacy and safety for romosozumab-to-denosumab were similar between Japanese women and the overall population. The sequence of romosozumab to rebuild the skeletal foundation before transitioning to antiresorptive treatment with denosumab is a promising regimen for Japanese postmenopausal women with osteoporosis at high risk of fracture.

**Electronic supplementary material:**

The online version of this article (10.1007/s11657-019-0608-z) contains supplementary material, which is available to authorized users.

## Introduction

Among postmenopausal women with osteoporosis, fractures are associated with substantial clinical and economic burden [1, 2]. A recent report estimated that although the incidence of hip fracture in Japan was decreasing, the prevalence was increasing as the number of elderly patients increased [3]. Key risk factors for fracture in postmenopausal women include low bone mineral density (BMD) and older age [4–14]. Fracture risk is particularly high in women with a prior history of fracture [15], yet globally, only about 20% of patients receive treatment to prevent recurrent fractures [16–18]. A study in Japan showed consistent findings, with only 19% of patients receiving pharmacologic medication in the year following a hip fracture [19].

In Japan, osteoporosis is a major public health problem, consistent with global trends. Approximately 13 million Japanese women have osteoporosis, 40% of whom are 70 years of age or older [20]. A threshold for intervention in Japanese guidelines for prevention and treatment of osteoporosis [21] is a 10-year probability of major osteoporotic fracture that is 15% or greater, based on the Fracture Risk Assessment Tool (FRAX) [22]. In 2010, an estimated 29.7 million women (46% of the total female population) in Japan were over the age of 50 years, and 9.3 million (31.5%) of those women exceeded the 15% threshold for intervention [23]. According to population growth estimation, the number of women aged 50 years or older above this threshold is projected to rise from 9.3 million to 12.7 million (38% of women over the age of 50 years) by the year 2035.

The most commonly prescribed initial therapy for the treatment of osteoporosis in Japan is an antiresorptive agent (oral bisphosphonate, selective estrogen receptor modulator, eldecalcitol, or denosumab) [24]. In Japan, two forms of teriparatide treatment are available: a subcutaneous injection of teriparatide acetate at 56.5 μg/week [25] and a subcutaneous injection of recombinant human teriparatide at 20 μg/day [13, 24]. A possible alternative for the treatment of osteoporosis is the bone-forming agent romosozumab, a monoclonal antibody that binds sclerostin, leading to the dual effect of increasing bone formation and decreasing bone resorption [26, 27]. In the FRActure study in postmenopausal woMen with ostEoporosis (FRAME; NCT01575834), a pivotal, international, double-blind, phase 3 study of postmenopausal women, 12 months of treatment with romosozumab once monthly, significantly improved BMD and reduced fracture risk vs placebo [28]. At 12 months, women in each treatment group transitioned to open-label treatment with the antiresorptive agent denosumab, administered every 6 months for an additional 24 months, including an open-label period (months 12–24) and an extension period (months 24–36). Significant improvements in BMD and fracture risk in the romosozumab-to-denosumab group persisted through 36 months in FRAME, supporting the benefit of rebuilding the skeletal foundation with romosozumab before transitioning to antiresorptive treatment with denosumab [29]. This subgroup analysis of FRAME examined cumulative results for BMD and fracture risk through 36 months among Japanese postmenopausal women with osteoporosis.

## Methods

The full methods for FRAME were reported previously [28]. Methods pertaining to the subgroup analysis are described here.

### Study participants

FRAME was an international study at 222 centers across 25 countries in North America, Europe, Central/South America, and Asia-Pacific (including 54 in Japan). Key inclusion criteria were postmenopausal women, age 55–90 years, and osteoporosis (BMD T-score − 3.5 to − 2.5 at total hip or femoral neck). Subjects had to have at least two vertebrae in the L1 through L4 region and at least one hip that could be evaluated by means of dual-energy x-ray absorptiometry (DXA). Due to the placebo-controlled study design, women were excluded from the study if they presented with a history of hip fracture, severe vertebral fracture, or more than two moderate vertebral fractures. Women with recent or repeated use of the following osteoporosis therapies were excluded from the study: strontium ranelate, fluoride, bisphosphonates, denosumab, any cathepsin K inhibitor, teriparatide, any parathyroid hormone analog, estrogen, hormonal ablation therapy, tibolone, cinacalcet, or calcitonin. Women with recent and prolonged use of systemic glucocorticoids were excluded from the study. Other key exclusion criteria were a history of metabolic bone disease or conditions affecting bone metabolism, osteonecrosis of the jaw, a 25-hydroxyvitamin D [25(OH)D] level of less than 20 ng/mL, and current hypercalcemia or hypocalcemia.

### Study design

During the 12-month double-blind period, subjects were randomized 1:1 to receive treatment with romosozumab 210 mg or placebo, administered subcutaneously once monthly (Online Resource 1). At 12 months, subjects in each treatment group transitioned to open-label treatment with denosumab 60 mg subcutaneously every 6 months for 24 months. Randomization was stratified by age (< 75 or ≥ 75 years) and prevalent vertebral fracture (yes or no). The original treatment assignment to romosozumab or placebo remained blinded throughout the study. All subjects received at minimum daily calcium (500–1000 mg) and vitamin D (600–800 IU) supplementation. If serum 25(OH)D was 20–40 ng/mL at baseline, then the subject could receive a vitamin D loading dose of 50,000–60,000 IU.

### Assessments

DXA scans of the lumbar spine and proximal femur were scheduled for all subjects at screening and at 12, 24, and 36 months. Lateral spine radiographs (thoracic and lumbar spine) were taken at screening and at 6, 12, 24, and 36 months, or when a subject experienced back pain suggestive of vertebral fracture. When a nonvertebral or clinical vertebral fracture was suspected, additional radiographs were obtained for confirmation. A central imaging vendor (BioClinica, Newark, CA, USA), blinded to the initial treatment assignment, assessed and graded vertebral radiographs (using the Genant semiquantitative criteria [30]) and nonvertebral radiographs. Only those fractures confirmed by the central imaging vendor were included for the analyses.

### Statistical analyses

For this report, most efficacy analyses were conducted in the Japanese full analysis set, defined as the subgroup of subjects at study centers in Japan who were randomized to treatment with romosozumab-to-denosumab or placebo-to-denosumab. The analysis set for new vertebral fractures at each visit included all randomized subjects who had a baseline and at least one postbaseline evaluation of vertebral fracture. The Japanese safety analysis set included all randomized subjects who received ≥ 1 dose of romosozumab or placebo in the 12-month double-blind period. Subject incidences of adverse events for the 36-month study period included all events that occurred in the double-blind period (among all subjects in the Japanese safety analysis set), as well as those in the open-label or extension periods (among those subjects who received ≥ 1 dose of denosumab).

Baseline demographics and clinical characteristics were summarized descriptively. The baseline 10-year probability of a major osteoporotic fracture was calculated with the FRAX algorithm including femoral neck BMD [22, 23]. Baseline BMD for each location was also expressed as the percentage of young adult mean. Diagnostic criteria for osteoporosis in Japan recommend calculation of the BMD as a percentage of the young adult mean value for adults 20–44 years of age (for lumbar spine) or 20–29 years of age (for the proximal femur) [31]. BMD values below 70% of the young adult mean in Japan correspond to approximately − 2.5 SD.

In FRAME, the primary efficacy endpoints were the incidence of new vertebral fracture through 12 and 24 months, and exploratory endpoints for the final analysis at 36 months included the incidences of new vertebral fracture and other fracture types, as well as the percentage change from baseline in BMD at the lumbar spine, total hip, and femoral neck. This subgroup analysis analyzed the same endpoints within the Japanese full analysis set. The key endpoint for this subgroup was percentage change from baseline of lumbar spine BMD. Fractures, including vertebral, clinical, and nonvertebral fractures, were also exploratory endpoints, but the subgroup analysis did not have adequate power to detect significant differences in endpoints. No multiplicity adjustment was applied. All *P* values were nominal.

Analyses of BMD used analysis of covariance models to determine the least squares mean percentage change from baseline, with adjustment for baseline BMD, machine type, and interaction between baseline BMD and machine type. A responder analysis assessed the percentage of subjects with a percentage change from baseline in BMD by DXA of varying magnitudes (i.e., ≥ 3%, ≥ 6%, and ≥ 10%, chosen empirically, with ≥ 3% representing the approximate least significant change) at the lumbar spine and total hip at 12 months, as well as subjects who had BMD decreases from baseline. The responder analysis used logistic regression models adjusting for treatment, age, and prevalent vertebral fracture stratification variables, baseline value, machine type, and baseline value-by-machine type interaction. Analyses of shifts in BMD T-score from ≤ − 2.5 at baseline to > − 2.5 at 12, 24, and 36 months at the lumbar spine and total hip used logistic regression models adjusting for treatment, age, prevalent vertebral fracture stratification variables, and baseline BMD T-score.

For new vertebral fracture, risk ratios were calculated by the Mantel-Haenszel method in the analysis set for new vertebral fractures, and *P* values were determined by logistic regression models that were stratified by age (< 75 or ≥ 75 years) and prevalent vertebral fracture (yes or no) at baseline. For other fracture types, hazard ratios and *P* values were determined by Cox proportional-hazards models that were also stratified by age and prevalent vertebral fracture. Nonvertebral fractures excluded fractures of the skull, facial bones, metacarpals, fingers, and toes; pathologic fractures; and fractures associated with high trauma. Major nonvertebral fractures included fractures of the pelvis, distal femur, proximal tibia, ribs, proximal humerus, forearm, and hip. Major osteoporotic fractures included clinical vertebral, hip, forearm, and humerus fractures, excluding pathologic fractures.

## Results

### Subject disposition

**Fig. 1:**
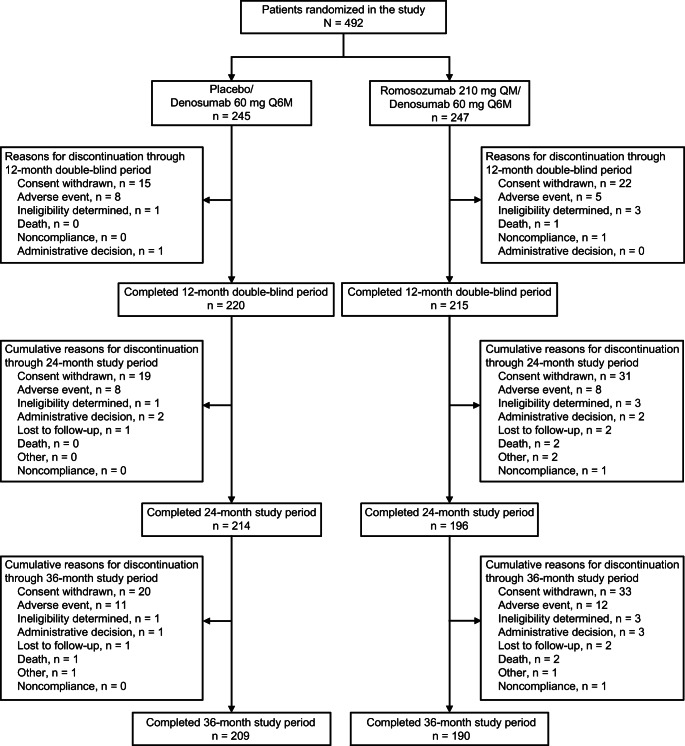
Subject disposition flowchart. *QM* once monthly, *Q6M* every 6 months

Japanese subjects were included in this subgroup analysis. The 7180 women who were randomized in FRAME were from the following regions: Central/Latin America (43.0%); Central and Eastern Europe (29.2%), Western Europe, Australia, or New Zealand (13.6%); Asia-Pacific (11.5%); and North America (2.7%). Of the 829 women from the Asia-Pacific region, most (59.3%) were from Japan (247 romosozumab, 245 placebo), followed by Hong Kong (27.9%) and India (12.8%). Overall, 81.1% (399/492) of Japanese women (190/247 romosozumab-to-denosumab vs 209/245 placebo-to-denosumab) completed the 36-month study period. The most common reason for early study discontinuation was consent withdrawn (33 (13.4%) romosozumab-to-denosumab vs 20 (8.2%) placebo-to-denosumab); other reasons such as adverse event (12 (4.9%) vs 11 (4.5%)), administrative decision (3 (1.2%) vs 1 (0.4%)), death (2 (0.8%) vs 1 (0.4%)), and lost to follow-up (2 (0.8%) vs 1 (0.4%)) had similar incidences in both treatment groups (Fig. 1).

### Baseline characteristics

Women in the romosozumab group were more likely than those in the placebo group to have at least one prevalent vertebral fracture (23.9% vs 18.8% of Japanese subjects), and these fractures were more likely to be moderate or severe (13.0% vs 6.9% of subjects; Table 1). Other baseline characteristics were similar between the romosozumab and placebo groups at baseline, including age, body mass index, BMD T-score (lumbar spine, total hip, and femoral neck), percentage of young adult mean, the incidence of nonvertebral fracture at age ≥ 45 years, and 25(OH)D. Median baseline FRAX scores for the 10-year probability of major osteoporotic fracture were also similar between the romosozumab and placebo groups, but the median baseline FRAX score overall in Japanese subjects was nearly double that in non-Japanese subjects.

**Table 1:** Baseline demographics and clinical characteristics

Characteristic	Placebo-to-denosumab (*N* = 245)	Romosozumab-to-denosumab (*N* = 247)
Age		
Years, mean (SD)	70.4 (6.6)	71.3 (6.8)
≥ 75 years, *n* (%)	68 (27.8)	86 (34.8)
Japanese, *n* (%)	245 (100.0)	247 (100.0)
Body mass index, kg/m^2^, mean (SD)	21.4 (2.8)	21.1 (2.9)
T-score,^a^ mean (SD)		
Lumbar spine	− 2.45 (0.82)	− 2.41 (0.90)
Total hip	− 2.44 (0.47)	− 2.44 (0.48)
Femoral neck	− 2.82 (0.30)	− 2.84 (0.30)
BMD % young adult mean, mean (SD)		
Lumbar spine	69.7 (9.9)	70.2 (10.9)
Total hip	68.0 (5.9)	68.0 (6.0)
Femoral neck	60.6 (4.0)	60.2 (4.1)
Prevalent vertebral fracture, *n* (%)		
0	193 (78.8)	186 (75.3)
1	35 (14.3)	47 (19.0)
≥2	11 (4.5)	12 (4.9)
Not readable/missing	6 (2.4)	2 (0.8)
Grade of most severe vertebral fracture,^b^ *n* (%)		
Mild	29 (11.8)	27 (10.9)
Moderate/severe	17 (6.9)	32 (13.0)
Nonvertebral fracture at ≥45 years of age, *n* (%)	60 (24.5)	53 (21.5)
FRAX 10-year probability of major osteoporotic fracture, median (IQR)	20.6 (14.8–27.9)	21.6 (15.5–26.8)
25-hydroxyvitamin D, ng/mL, median (IQR)	28.6 (23.8–33.8)	29.6 (24.0–35.2)

*BMD* bone mineral density, *FRAX* Fracture Risk Assessment Tool [22], *IQR* interquartile range, *N* number of subjects randomized, *SD* standard deviation

^a^Derived from Japanese reference ranges

^b^The most severe vertebral fracture was assessed with the use of the Genant semiquantitative grading scale

### Bone mineral density

**Fig. 2:**
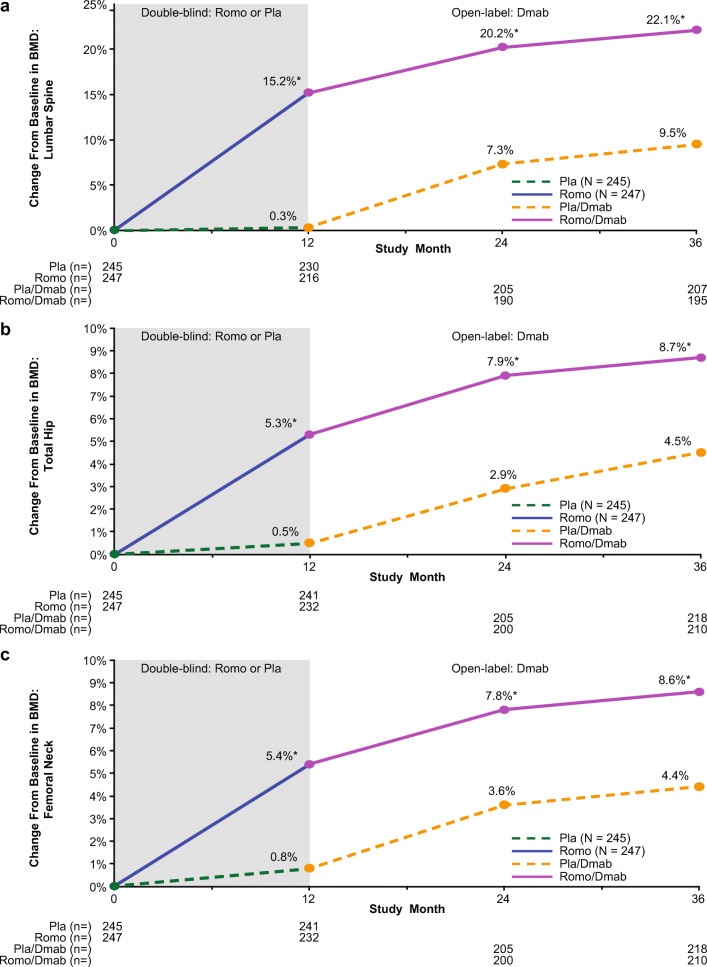
Percentage change from baseline in bone mineral density (BMD): effect of romosozumab (Romo) treatment for 12 months followed by denosumab (Dmab) treatment for 24 months. Least squares mean percentage changes at **a** lumbar spine, **b** total hip, and **c** femoral neck. *n* number of subjects with evaluable data at the time point, *N* number of subjects randomized, *Pla* placebo, *QM* once monthly, *Q6M* every 6 months. *Nominal *P* < 0.001 between treatment groups based on analysis of covariance model adjusting for treatment, age, prevalent vertebral fracture stratification variables, baseline value, machine type, and baseline value-by-machine type interaction

After 12 months of romosozumab treatment, the mean increase in BMD from baseline was 15.2% at the lumbar spine (Fig. 2a), 5.3% at the total hip (Fig. 2b), and 5.4% at the femoral neck (Fig. 2c). Each of these increases was significantly different (*P* < 0.001) from the mean changes that occurred at 12 months in the placebo group (lumbar spine, 0.3%; total hip, 0.5%; and femoral neck, 0.8%). Even after subjects transitioned to denosumab, the percentage increases in BMD from baseline continued to favor the romosozumab-to-denosumab group over the placebo-to-denosumab group through 24 and 36 months. The mean increases in BMD at 36 months in the romosozumab-to-denosumab vs placebo-to-denosumab groups, respectively, were lumbar spine, 22.1% vs 9.5% (difference, 12.7%; *P* < 0.001); total hip, 8.7% vs 4.5% (difference, 4.2%; *P* < 0.001); and femoral neck, 8.6% vs 4.4% (difference, 4.1%; *P* < 0.001).

**Fig. 3:**
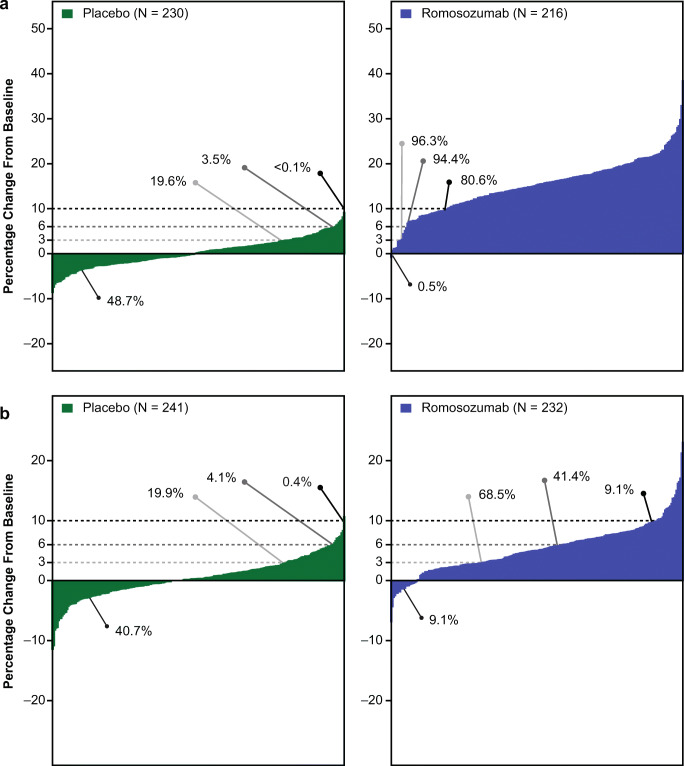
Responder analysis of percentage change from baseline to 12 months in bone mineral density (BMD) for individual subjects. **a** Lumbar spine. **b** Total hip. The *x* axis represents each individual subject. Horizontal lines reflect 3%, 6%, and 10% responses relative to baseline. Arrowheads and values represent the percentage of subjects with the indicated percentage changes in BMD. *N* number of subjects with a baseline BMD assessment and at least one postbaseline BMD assessment at or before 12 months

In the responder analysis after 12 months of treatment, lumbar spine BMD gains of ≥ 3%, ≥ 6%, and ≥ 10% from baseline, respectively, were achieved by 96.3%, 94.4%, and 80.6% of subjects in the romosozumab group and by 19.6%, 3.5%, and < 0.1% of subjects in the placebo group (Fig. 3a). Total hip BMD gains of ≥ 3%, ≥ 6%, and ≥ 10%, respectively, were achieved by 68.5%, 41.4%, and 9.1% of subjects in the romosozumab group and by 19.9%, 4.1%, and 0.4% of subjects in the placebo group (Fig. 3b). Each of the response rates for BMD was significantly greater in the romosozumab group than in the placebo group (*P* < 0.001).

Of the subjects with a lumbar spine T-score of ≤ − 2.5 at baseline, a T-score of > − 2.5 at 12, 24, and 36 months, respectively, was observed for 67.3%, 85.7%, and 89.9% of subjects in the romosozumab-to-denosumab group and for 10.3%, 34.6%, and 43.4% of subjects in the placebo-to-denosumab group. Of the subjects with a total hip T-score of ≤ − 2.5 at baseline, a T-score of > − 2.5 at 12, 24, and 36 months, respectively, was observed for 42.7%, 58.5%, and 65.3% of subjects in the romosozumab-to-denosumab group and for 16.4%, 36.4%, and 44.9% of subjects in the placebo-to-denosumab group. At each assessment, the percentage of subjects with a positive shift in T-score was significantly greater in the romosozumab-to-denosumab group than in the placebo-to-denosumab group (*P* < 0.001).

### Fracture risk

**Fig. 4:**
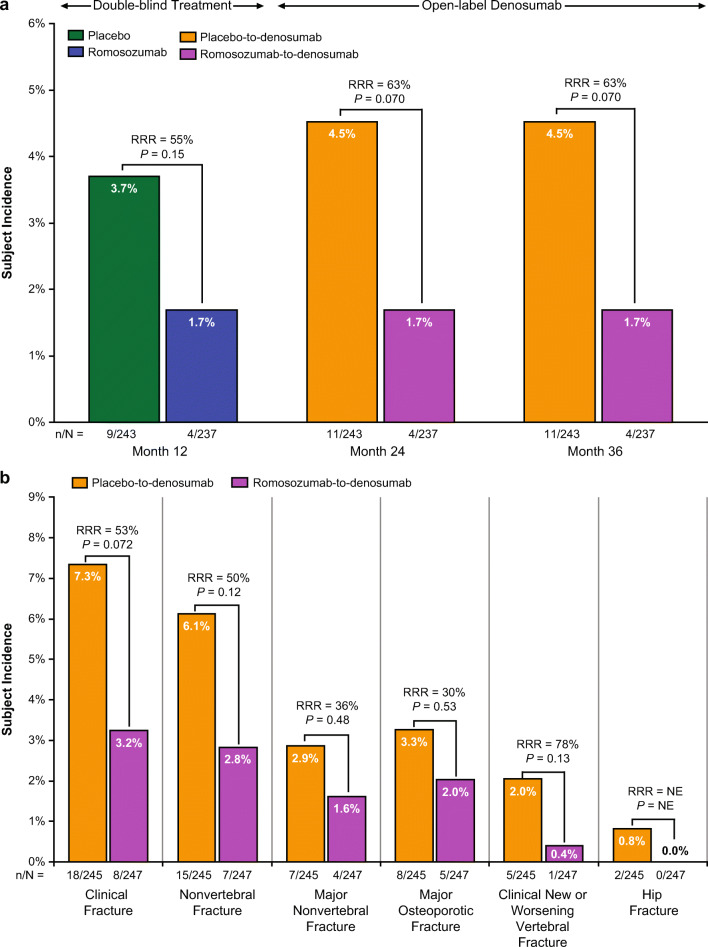
Fracture risk in Japanese postmenopausal women: effect of romosozumab or placebo for 12 months, followed by denosumab treatment in all subjects for 24 months. **a** Subject incidence and relative risk reduction (RRR), based on relative risks, for new vertebral fracture by study visit in the analysis set for vertebral fractures. **b** Subject incidence and RRR, based on hazard ratios, for key fracture endpoints in the full analysis set through 36 months. The last observation was carried forward for missing data. *n* number of subjects with fracture, *N* number of subjects analyzed, *NE* not evaluable

Romosozumab treatment for 12 months was associated with a 55% lower risk of new vertebral fracture compared with placebo (*P* = 0.15; Fig. 4a). After all subjects transitioned to open-label denosumab treatment, romosozumab-to-denosumab had a lower risk of new vertebral fracture by 63% at both 24 and 36 months compared with placebo-to-denosumab (*P* = 0.070). The cumulative subject incidences of new vertebral fracture were 1.7% (4/237) at each time point in the romosozumab-to-denosumab group and 3.7% (9/243), 4.5% (11/243), and 4.5% (11/243) at 12, 24, and 36 months, respectively, in the placebo-to-denosumab group.

The risk for fracture by 36 months was lower in the romosozumab-to-denosumab group compared with the placebo-to-denosumab group by 30–78% for each fracture type examined (clinical (*P* = 0.072), nonvertebral (*P* = 0.12), major nonvertebral (*P* = 0.48), major osteoporotic (*P* = 0.53), and clinical new or worsening vertebral (*P* = 0.13); Fig. 4b). The risk for hip fracture was lower as well (0.8 vs 0.0%), but as no subject in the romosozumab-to-denosumab group had a hip fracture, no risk reduction or *P* value could be calculated. The subject incidence of fracture through 36 months was numerically lower in the romosozumab-to-denosumab group than in the placebo-to-denosumab group for all fracture types examined.

In the Kaplan-Meier time-to-event analyses for clinical fractures and nonvertebral fractures, cumulative fracture rates were lower through follow-up in the romosozumab-to-denosumab group than in the placebo-to-denosumab group (Online Resource 2). For each of these fracture types, separation of the curves for fracture incidence appeared within the first few months and the separation of the curves increased over time, even after all subjects had transitioned to denosumab treatment.

### Safety

The Japan safety analysis set included all 489 subjects in Japan (245 romosozumab, 244 placebo) who received at least one dose of study treatment. The incidences of adverse events through 36 months were overall balanced between the romosozumab-to-denosumab and placebo-to-denosumab groups (87.8% vs 89.8%; Table 2). The most commonly reported adverse events, which occurred with similar incidences in each group, were nasopharyngitis, back pain, fall, osteoarthritis, contusion, and constipation. Adverse events led to study drug discontinuation for 4.5% of subjects in the romosozumab-to-denosumab group and 4.9% of subjects in the placebo-to-denosumab group.

**Table 2:** Subject incidence of treatment-emergent adverse events through 36 months

	Placebo-to-denosumab (*N* = 244)	Romosozumab-to-denosumab (*N* = 245)
Any adverse event	219 (89.8)	215 (87.8)
Most frequent adverse events^a^		
Nasopharyngitis	91 (37.3)	103 (42.0)
Back pain	31 (12.7)	35 (14.3)
Fall	32 (13.1)	30 (12.2)
Osteoarthritis	30 (12.3)	29 (11.8)
Contusion	26 (10.7)	29 (11.8)
Constipation	24 (9.8)	28 (11.4)
Any serious adverse event	37 (15.2)	39 (15.9)
Any serious cardiovascular adverse event^b^	2 (0.8)	3 (1.2)
Death	1 (0.4)	2 (0.8)
Cardiovascular death^b^	1 (0.4)	2 (0.8)
Leading to study drug discontinuation	12 (4.9)	11 (4.5)
Leading to study discontinuation	11 (4.5)	12 (4.9)
Events of interest^c^		
Hypersensitivity^d^	54 (22.1)	46 (18.8)
Osteoarthritis	47 (19.3)	51 (20.8)
Hyperostosis	10 (4.1)	18 (7.3)
Malignancy	4 (1.6)	9 (3.7)
Injection-site reaction	3 (1.2)	8 (3.3)
Osteonecrosis of the jaw^b^	0 (0.0)	1 (0.4)
Atypical femoral fracture^b^	0 (0.0)	0 (0.0)
Hypocalcemia	0 (0.0)	0 (0.0)

*N* number of subjects who were randomized and received at least one dose of the study drug in the 12-month double-blind study period. The subject incidence rates include all events that occurred in the 12-month double-blind period and, in addition, all events that occurred in the open-label and extension periods for those subjects who received at least one dose of denosumab

^a^Most frequent adverse events occurring in ≥ 10% of subjects in either treatment group

^b^Includes adverse events adjudicated positive by an independent adjudication committee. For cardiovascular deaths, includes fatal events adjudicated as cardiovascular-related or undetermined (presumed cardiac-related)

^c^Identified by prespecified Medical Dictionary for Regulatory Activities (MedDRA) search strategies using MedDRA version 19.1. Hypocalcemia, injection-site reaction, osteoarthritis, and hyperostosis include only treatment-emergent adverse events as a result of Amgen-defined MedDRA search strategies. Hypersensitivity and malignancy include only treatment-emergent adverse events as a result of a narrow search/scope in standardized MedDRA queries

^d^Serious adverse events of hypersensitivity were reported in 1 subject who received romosozumab followed by denosumab occurring in the first 12 months and in 0 subjects who received placebo followed by denosumab

The subject incidence of any serious adverse event was 15.9% for romosozumab-to-denosumab and 15.2% for placebo-to-denosumab. Positively adjudicated cardiovascular death was reported for 2 (0.8%) subjects in the romosozumab-to-denosumab group (1 with congestive cardiomyopathy during the double-blind period and 1 with multiple organ dysfunction syndrome after the transition to denosumab) and 1 (0.4%) subject in the placebo-to-denosumab group (acute cardiac failure after the transition to denosumab). Investigators did not consider any of the fatal events to be related to study treatment. No other deaths were reported. Positively adjudicated nonfatal cardiovascular events were reported for 2 (0.8%) subjects in the romosozumab-to-denosumab group (1 with acute myocardial infarction during the double-blind period and 1 with cerebral hemorrhage after the transition to denosumab (this subject also had the fatal multiple organ dysfunction syndrome)) and 1 (0.4%) subject in the placebo-to-denosumab group (cerebral infarction during the double-blind period).

The subject incidences for adverse events of interest in the romosozumab-to-denosumab and placebo-to-denosumab groups were as follows: hypersensitivity (18.8% vs 22.1%), osteoarthritis (20.8% vs 19.3%), hyperostosis (7.3% vs 4.1%), malignancy (3.7% vs 1.6%), and injection-site reaction (3.3% vs 1.2%). Positively adjudicated osteonecrosis of the jaw occurred in 1 (0.4%) subject in the romosozumab-to-denosumab group and no subjects in the placebo-to-denosumab group. This case occurred after 12 months of romosozumab treatment and one dose of denosumab, following a tooth extraction and subsequent osteomyelitis of the jaw. No subject had a positively adjudicated atypical femoral fracture or hypocalcemia. Adverse events of hypersensitivity and injection-site reaction were mostly mild in severity, and none led to discontinuation of treatment in the romosozumab-to-denosumab group.

Binding antibodies to romosozumab were observed at any time for 58 (23.7%) subjects in the romosozumab-to-denosumab group. Neutralizing antibodies to romosozumab developed at any time for 2 (0.8%) subjects in the romosozumab-to-denosumab group. Antibodies to romosozumab had no detectable effect on efficacy or safety.

## Discussion

In this subgroup analysis of Japanese postmenopausal women with osteoporosis in the phase 3 FRAME trial, the outcomes for BMD, fracture risk, and safety through 36 months were consistent with those for the overall FRAME population [28, 29]. In both populations, subjects in the romosozumab-to-denosumab group showed significantly higher increases in BMD at the spine, total hip, and femoral neck at 12, 24, and 36 months than the subjects in the placebo-to-denosumab group, and the romosozumab-to-denosumab group had consistently lower fracture incidences for all fracture types evaluated. Compared with the overall population of FRAME, Japanese women had lower mean body mass index, higher prevalence of previous nonvertebral fracture at ≥ 45 years of age, and nearly double the median baseline FRAX 10-year probability of major osteoporotic fracture. Baseline T-scores and percentage changes from baseline for BMD at 12, 24, and 36 months at the total hip and femoral neck were similar between Japanese subjects and the overall FRAME population. Reference values for peak bone mass or young adult mean are lower in Japanese women than in Caucasian women, so the same T-score corresponds to a lower baseline BMD in Japanese women.

BMD responder analyses and T-score shift analyses provided further evidence of the consistent bone-forming effect of romosozumab among Japanese postmenopausal women with osteoporosis. By 12 months, a meaningful gain of ≥ 3% for BMD at the lumbar spine was achieved by almost every woman in the romosozumab group and less than 20% of women in the placebo group; this included substantial gains of ≥ 10% in BMD at the lumbar spine for > 80% of women in the romosozumab group and < 1% of women in the placebo group. A working group from the American Society for Bone and Mineral Research and the United States National Osteoporosis Foundation developed principles of goal-directed treatment for osteoporosis that included freedom from fracture and a T-score > −2.5 [32]. In this study, romosozumab was associated with positive shifts at the lumbar spine from a T-score of ≤ − 2.5 at baseline to > − 2.5 at 12 months for more than 2 in 3 women, compared with approximately 1 in 10 women in the placebo group. Thus, as a result of the observed increases in BMD during romosozumab treatment, only one-third of Japanese subjects in the romosozumab group (vs 90% of subjects in the placebo group) still met the lumbar spine T-score criterion for osteoporosis at 12 months, when they transitioned to denosumab. Progressively, more women in each treatment group who started the study with a T-score of ≤ − 2.5 at baseline shifted to a T-score of > − 2.5 at 24 and 36 months. These findings suggest that romosozumab followed by denosumab is a promising treatment regimen to achieve treatment goals in Japanese postmenopausal women with osteoporosis.

As was observed in the overall population [28, 29], Japanese women in FRAME who received romosozumab during the first 12 months of the study had a lower risk of new vertebral fracture compared with women who received placebo during the first 12 months, but the subgroup analysis did not have adequate power to demonstrate statistically significant reductions in fracture risk. The relative risk reduction for this endpoint in the romosozumab-to-denosumab group compared with the placebo-to-denosumab group was 73% overall and 55% in Japanese women, reflecting similar trends. In both populations, the benefits for vertebral fractures persisted at 24 and 36 months, after all subjects from each treatment group transitioned to open-label antiresorptive treatment with denosumab. In each analysis, the romosozumab-to-denosumab group had lower subject incidences than the placebo-to-denosumab group at 36 months for all other fracture types examined. This study evaluated clinical fractures (including nonvertebral fractures and clinical vertebral fractures, defined as radiographically confirmed new or worsening vertebral fractures associated with back pain), nonvertebral fractures, and other fracture types (including major nonvertebral, major osteoporotic, clinical new or worsening vertebral, and multiple new or worsening vertebral fractures). In the overall FRAME population, risk reductions for fractures at 36 months favored the romosozumab-to-denosumab group over the placebo-to-denosumab group. Among Japanese women, the risk for nonvertebral fractures was 50% lower in the romosozumab-to-denosumab group, which did not achieve statistical significance.

In Japanese women, adverse events were generally similar between the treatment groups and were consistent with those of the overall FRAME population [28], including subject incidences of all adverse events, serious adverse events, adjudicated cardiovascular events, deaths, adjudicated cardiovascular deaths, events leading to study discontinuation, the most commonly reported events, and events of interest, which include hypocalcemia, hypersensitivity, injection-site reactions, malignancy, osteoarthritis, hyperostosis, atypical femoral fracture, and osteonecrosis of the jaw. Incidences of positively adjudicated cardiovascular events, deaths, and positively adjudicated cardiovascular deaths were low and similar between the romosozumab-to-denosumab and placebo-to-denosumab groups in Japanese subjects, as they were in the overall FRAME population. One case of osteonecrosis of the jaw was reported in a Japanese woman after romosozumab treatment and the first dose of denosumab, following a tooth extraction and subsequent osteomyelitis of the jaw. Antibodies to romosozumab had no detectable effect on efficacy or safety among Japanese women.

The main limitation of this analysis was the examination of a subgroup of subjects from an international study that was neither designed nor powered to demonstrate the safety and efficacy of the study treatment in this subgroup.

While a majority of Japanese postmenopausal women with osteoporosis receive an antiresorptive agent as their initial treatment [24], the available evidence from global clinical research supports rebuilding the skeletal foundation with romosozumab, followed by antiresorptive therapy with denosumab. This treatment sequence offers patients rapid BMD increases and fracture reduction benefits that are sustained over time [7, 33]. In conclusion, outcomes for romosozumab-to-denosumab through 36 months in Japanese subjects were consistent with those in the overall FRAME population. Romosozumab, as a foundational bone-forming treatment, followed by denosumab, is a promising regimen for Japanese postmenopausal women with osteoporosis at high risk of fracture.

## Electronic Supplementary Material

ESM 1 (DOCX 183 kb)

## Data Availability

Qualified researchers may request data from Amgen clinical studies. Complete details are available at the following: https://wwwext.amgen.com/science/clinical-trials/clinical-data-transparency-practices/.
