# Supplementary material for: Increased bone mineral density for 1 year of romosozumab, vs placebo, followed by 2 years of denosumab in the Japanese subgroup of the pivotal FRAME trial and extension
Source: Arch Osteoporos. 2019 Jun 5;14(1):59. doi: 10.1007/s11657-019-0608-z (PMC6551345; doi:10.1007/s11657-019-0608-z)
Supplement: Supplementary file 1 — (DOCX 183 kb) [file 11657_2019_608_MOESM1_ESM.docx]

**Electronic supplementary material**

**Increased bone mineral density and lower fracture risk for 1 year of romosozumab, vs placebo, followed by 2 years of denosumab in the Japanese subgroup of the pivotal FRAME trial and extension**

Akimitsu Miyauchi • Rajani V. Dinavahi • Daria B Crittenden • Wenjing Yang • Judy C. Maddox • Etsuro Hamaya • Yoichi Nakamura • Cesar Libanati • Andreas Grauer • Junichiro Shimauchi

**Correspondence:** Akimitsu Miyauchi, Miyauchi Medical Center, Osaka, Japan; [akimiyauchi0129@gmail.com](mailto:akimiyauchi0129@gmail.com); Tel: +81 72 686 3330; Fax: +81 72 686 3331

**Online Resource 1**

**Study design.** Women were randomly assigned, in a 1:1 ratio, to receive subcutaneous (SC) injections of 210 mg of romosozumab or placebo SC once monthly (QM) for 12 months during the double-blind phase of the trial. Subjects then received open-label denosumab 60 mg SC every 6 months (Q6M) for an additional 12 months (open-label period). Women who received denosumab at 12 and 18 months and who completed the 24-month study period continued to receive open-label denosumab (60 mg SC Q6M) in a 12-month extension period. The initial group assignment was still blinded to the investigator and subjects during the open-label and extension periods. Subjects were stratified at randomization according to age (<75 years vs ≥75 years) and prevalent vertebral fracture (yes vs no). ^a^ At day 0, subjects with serum 25(OH)D 20–40 ng/mL at baseline could receiving a loading dose of 50,000–60,000 IU vitamin D


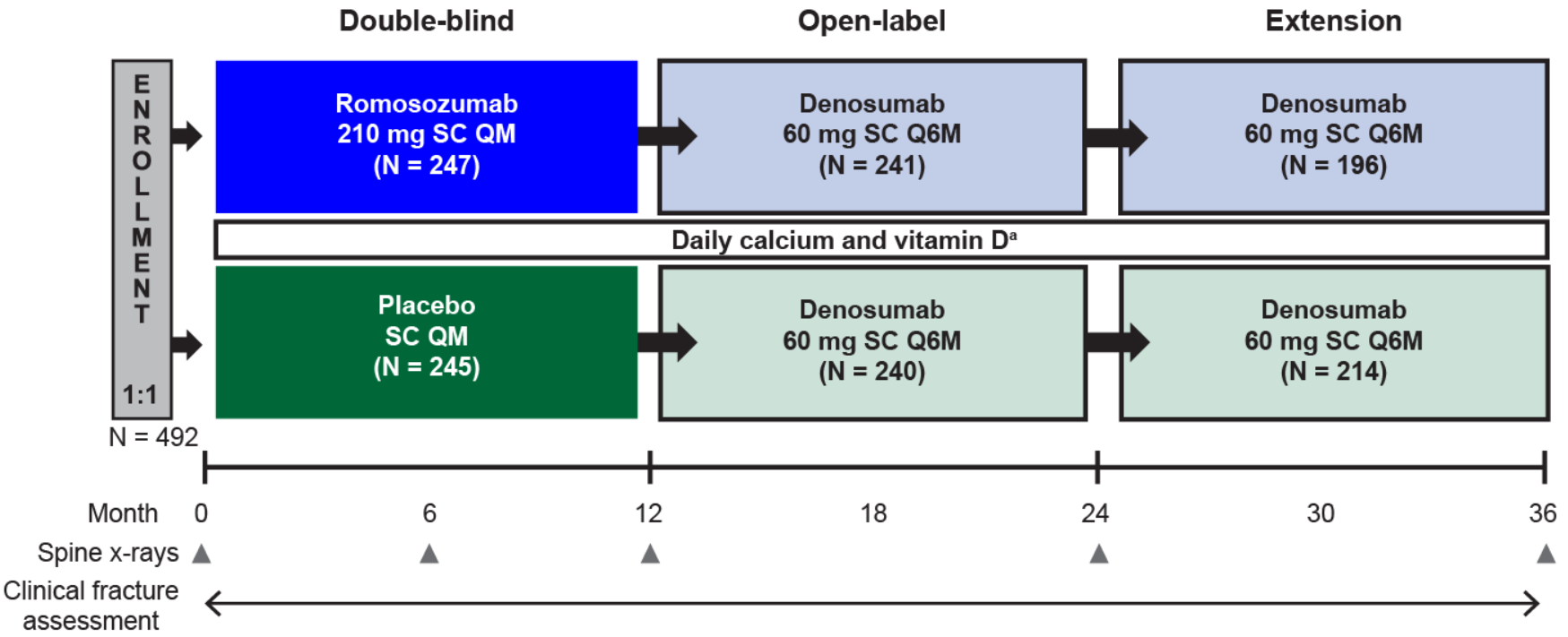


**Online Resource 2**

Bone mineral density at baseline, by machine type

|  |  |  | Placebo-to- denosumab | Romosozumab-to- denosumab |
| --- | --- | --- | --- | --- |
| Lumbar spine, g/cm^2^ | Lunar | *n* | 9 | 10 |
|  |  | Mean (SD) | 0.756 (0.118) | 0.809 (0.151) |
|  | Hologic | *n* | 221 | 207 |
|  |  | Mean (SD) | 0.692 (0.098) | 0.694 (0.107) |
| Total hip, g/cm^2^ | Lunar | *n* | 12 | 13 |
|  |  | Mean (SD) | 0.612 (0.029) | 0.586 (0.050) |
|  | Hologic | *n* | 229 | 221 |
|  |  | Mean (SD) | 0.597 (0.052) | 0.598 (0.051) |
| Femoral neck, g/cm^2^ | Lunar | *n* | 12 | 13 |
|  |  | Mean (SD) | 0.580 (0.033) | 0.566 (0.061) |
|  | Hologic | *n* | 229 | 221 |
|  |  | Mean (SD) | 0.478 (0.032) | 0.476 (0.032) |

**Online Resource 3**

Time to fracture in Japanese postmenopausal women: effect of romosozumab or placebo for 12 months, followed by denosumab (Dmab) treatment in each group for 24 months. **a** Time to first clinical fracture. **b** Time to first nonvertebral fracture. *N* number of subjects randomized
